# Retinal-image quality and contrast sensitivity function in eyes with epiretinal membrane: a cross-sectional observational clinical study

**DOI:** 10.1186/s12886-018-0957-1

**Published:** 2018-11-07

**Authors:** Limei Liu, Yi Wang, Ju Liu, Wu Liu

**Affiliations:** 1 Beijing Tongren Eye Center, Beijing Tongren Hospital, Capital Medical University, Beijng Ophthalmology and Visual Sciences Key Laboratory, Beijing, China; 2 Department of Ophthalmology, Yantai Yuhuangding Hospital, Affiliated Hospital of Medical College, Qingdao University, Yantai, Shandong China

**Keywords:** Epiretinal membrane, Retinal-image quality, Visual performance

## Abstract

**Background:**

To investigate the effect of idiopathic epiretinal membrane (ERM) on the retinal-image quality and psychophysical contrast sensitivity function (CSF).

**Methods:**

Forty-four subjects with diagnosis of idiopathic unilateral ERM were enrolled in this cross-sectional observational clinical study. The fellow unaffected eyes were set as the control group. For retinal-image quality assessment, an Optical Quality Analysis System (OQAS) based on double-pass technique was used to evaluate objective scatter index (OSI) and Strehl ratio. For visual performance, the CSF under photopic condition was measured.

**Results:**

For retinal-image quality, the result of double-pass device revealed a significant lower Strehl ratio and larger OSI in the ERM eyes compared to the fellow eyes (all *P* < 0.05). For visual performance, the CSF at all spatial frequencies under photopic condition were also significantly degraded in the ERM eyes compared to the fellow eyes (all *P* < 0.05). For the ERM eyes, the reduction of Strehl ratio and CSF was 29.41 and 54.39%, respectively, and the increase of OSI was 164.10% compared to the fellow eyes. Besides, BCVA significantly correlated to the total CSF (ERM eyes, *r* = − 0.53, *P* < 0.001; the fellow eyes, *r* = − 0.467, *P* = 0.002) and Strehl ratio (ERM eyes, *r* = − 0.485, *P* = 0.001; the fellow eyes, *r* = − 0.311, *P* = 0.043) in both of the ERM and the fellow eyes.

**Conclusion:**

Eyes affected with ERM showed poorer retinal-image quality and visual performance than the normal eyes. Retinal-image quality measured by OQAS based on double-pass technique could be useful for assessing the retinal-image quality for ERM-affected eyes, in which retinal scattering was significantly increased.

**Electronic supplementary material:**

The online version of this article (10.1186/s12886-018-0957-1) contains supplementary material, which is available to authorized users.

## Background

Epiretinal membrane (ERM) is a common retinal disease leading to significant visual impairment with an incidence of 2.2 and 28.9% [1–3]. In addition to the secondary ERM which can be caused by ocular pathology such as inflammation, trauma, tumors, or intraocular surgery, idiopathic ERM formation is a primary disease which can also often occur in the elderly [4]. It is characterized by a semi-translucent, glial and fibrocellular proliferative membrane at the vitreoretinal interface [5], which can exert tractional forces causing displacement of retina and retinal vessels [6, 7]. This pathology with characteristic morphological abnormalities, including curling and/or straightening of retinal vessels and deformation or displacement of the retinal tissues, may have a negative impact on the visual quality by changing the light reflected onto the altered retina.

For vision assessment, visual acuity (VA) is the most widely used parameter, but it cannot completely represent the whole visual performance at different degrees of contrast and spatial frequency in the real world. OQAS based on the double-pass technique has been used in the past to detect the retinal-image quality, such as optical aberration, diffraction and scattering, with good reliability [8–11]. The OQAS can provide parameters such as objective scatter index (OSI), Strehl ratio and modulation transfer function cut off requency (MTF cutoff), and it has been used extensively for visual asthenopia [8], eyes implanted with diffractive multifocal or monofocal intraocular lenses [9], endothelial keratoplasty [10] and healthy young population [11]. Contrast sensitivity function (CSF), providing the ability to detect difference in luminance and distinguishing details, is another good choice which can provide more information on visual function than VA [12]. It has been revealed that impaired CS may be found in cases of normal VA [13].

Although the superiority of retinal-image quality and CSF for assessing the visual function has been demonstrated and used in series of researches about many kinds of patients or healthy people, there is still a lack of information on the difference of retinal-image quality and psychophysical CSF between the ERM and healthy eyes. In this study, objective parameters of optical quality, including objective scatter index (OSI), Strehl ratio, and psychophysical CSF at five spatial frequencies (1.5, 3, 6, 12, and 18 cpd (cycles per degree)) were used to assess the effect of retinal structural change on the visual function in the ERM eyes.

## Methods

This cross-sectional observational clinical study on patients with idiopathic unilateral ERM was conducted in the Beijing Tongren Hospital between October 2013 and July 2014. This study followed the tenets of the Declaration of Helsinki, and was approved by the ethical committee of the Beijing Tongren Hospital. Informed consent was obtained from each patient before the study.

The inclusion criterion was the presence of unilateral ERM. The exclusion criterion included: history of any intraocular surgery except for uncomplicated phacoemulsification, presence or history of age-related macular degeneration, diabetic retinopathy, retinal detachment, central or branch retinal vein occlusion, central or branch retinal artery occlusion, inflammatory eye disorders, different type and severity of cataract between the two eyes, ocular trauma, or any other potential cause of vision loss than ERM.

All the patients were assessed by two independent observers. Cycloplegic manifest refractions were conducted, and the best corrected VA (BCVA) was recorded as logMAR values for statistical analysis. LOCS III slitlamp grading was used to match the type and severity of cataract between the ERM and the fellow eyes.

A high-definition optical coherence tomography (Cirrus HD-OCT, Carl-Zeiss Meditec, Dublin, CA, USA) was conducted with a macular cube 512 × 128 combo across an area of 6 × 6 mm. The stages of ERM were identified with B-scan OCT images according the indication of Govetto et al. [14]:stage I: presence of mild ERM (the morphologic or anatomic disruption was negligible); stage II: the foveal depression was absent and the outer nuclear layer was characteristically stretched; stage III: the inner foveal layers anomalously crossed the central foveal area with a less pronounced widening of the outer nuclear layer, but all the retinal layers could be clearly identified on the OCT images; stage IV: the disruption of the macula was remarkable and the retinal layers could not be clearly identified on the OCT images.

Data of objective visual quality (retinal-image quality) were taken using an optical-quality device, OQAS (Optical Quality Analysis System, Visiometrics SL, Tarrasa, Spain), based on the double-pass technique. Spherical error was automatically corrected, and the cylindrical error was corrected with appropriate lens in order to optimize measurements on the double-pass system. In order to minimize the effect of tear film on the light scattering, all the measurements were taken after several eye blinks. The optical parameters including OSI and Strehl ratio were recorded for 4-mm pupil. The OSI parameter is often used to qualify the intraocular scattered light [15] that a small OSI value is usually correlated to eyes with low scattering. The Strehl ratio is often computed as the ratio between MTF area of the eye and the diffraction-limited MTF area. For normal people, it is about 30% [16], and a lower Strehl ratio value indicates a poor optical system aberration.

The Optec 6500 vision testing system (Stereo Optical Co. Inc., Chicago, IL, USA) was used to measure the functional acuity contrast testing (F.A.C.T). Tests were performed monocularly with best spectacle correction and natural pupil. The sinusoidal grating was set as the target chart and located at a fixed distance (2.5 m from the subjects) under a constant luminance of 85 cd/m^2^. CS values were converted to numerical values at five spatial frequencies (1.5, 3, 6, 12, and 18 cpd) by using a conversion chart of F.A.C.T. All the measurements were conducted three times at all spatial frequencies to confirm an appropriate result.

For statistical analysis, SPSS Version 16.0 (SPSS 16.0, Inc., Chicago, IL) was used. Firstly, a Sample K-S test was used to test the data for normality. A paired-samples t test was used to compare the values of spherical equivalent refractive error (SER), BCVA, severity of cataract, parameters of retinal-image quality and CSF between the ERM and fellow eyes. Characteristics, optical performance and CSF in the 4 subgroups of the ERM eyes were compared using a one-way ANOVA. The CSF as a function of the Strehl ratio was analyzed to identify the correlation between visual performance and retinal-image quality using Pearson correlation analysis. The association between the BCVA with the total CSF and Strehl ratio was also tested using Pearson correlation analysis. For ERM eyes, association between the severity of ERM with BCVA, OSI, Strehl ratio and total CSF was assessed by excluding the effect of the grade of cataract using partial correlation analysis. *P* values lower than 0.05 at two tails were considered statistically significant.

## Results

In all, 44 patients with unilateral ERM (15 males and 29 females) were enrolled in this study. The average age of patients was 63.70 ± 8.38 years (range from 38 to 74 years). The mean grade of LOCS III nuclear color (NC), nuclear opalescence (NO), cortical cataract (C) and posterior subcapsular cataract (P) was 2.80 ± 0.94, 2.56 ± 0.92, 0.61 ± 0.87 and 0.05 ± 0.21, respectively.

**Fig. 1:**
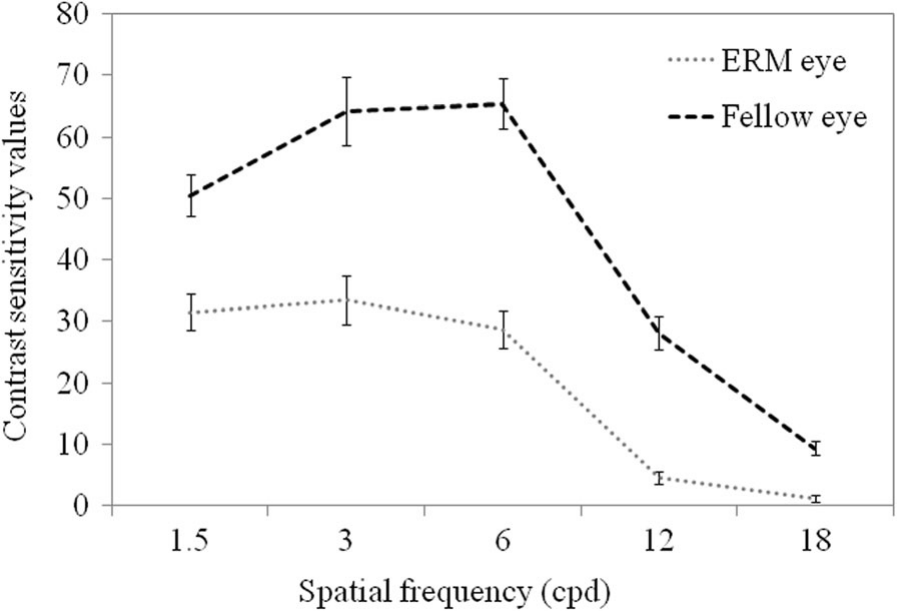
Average CSF at five spatial frequencies for the ERM eyes and the fellow healthy Eyes. Data includes standard error

Table 1 summarizes the results of Paired-samples t test comparing the ERM eyes with the fellow eyes. The refraction error was best matched between the ERM and fellow eyes. Visual acuities accessed by logMAR of the ERM eyes were significantly worse than that of the fellow eyes (0.48 ± 0.28 vs. 0.02 ± 0.14, *P* < 0.001). Two subjects have accepted bilateral cataract surgery and intraocular lens implant, and the type and severity of cataract classified using LOCS III slitlamp grading system were similar between the two eyes of all the included patients (all *P* > 0.05). Retinal-image quality accessed by OQAS of the ERM eyes was worse than that of the fellow eyes. For the ERM eyes, values of OSI was significantly larger, and values of the Strehl ratio were significantly less compared to the fellow eyes (all *P* < 0.05). In addition, for the ERM eyes, the reduction of Strehl ratio was 29.41%, and the increase of OSI was 164.10% compared to the fellow eyes. When compared to the fellow eyes, the CS values of the ERM eyes were significantly decreased at all spatial frequencies (1.5, 3, 6, 12, 18 cpd), under photopic condition (all *P* < 0.05, Fig. 1 & Table 2). Reduction of the total CS values was 54.39% for the ERM eyes.

**Table 1:** Difference between ERM eyes and the fellow eyes at presentation

Parameters	ERM eyes	Fellow eyes	*P*-value
SER (D)	0.08 ± 1.24	−0.09 ± 1.23	0.306
BCVA (logMAR)	0.48 ± 0.28	0.02 ± 0.14	< 0.001
LOCS III NC grade	2.80 ± 0.94	2.75 ± 0.87	0.486
LOCS III NO grade	2.56 ± 0.92	2.54 ± 0.89	0.781
LOCS III C grade	0.61 ± 0.87	0.65 ± 0.74	0.511
LOCS III P grade	0.05 ± 0.21	0.07 ± 0.25	0.323
OSI	3.09 ± 1.90	1.17 ± 1.11	< 0.001
Strehl ratio	0.12 ± 0.05	0.17 ± 0.05	< 0.001
Spatial frequency (cpd)			
1.5	31.55 ± 19.48	50.45 ± 22.38	< 0.001
3	33.52 ± 26.07	64.23 ± 36.89	< 0.001
6	28.73 ± 20.62	65.41 ± 26.44	< 0.001
12	4.61 ± 6.79	28.16 ± 18.72	< 0.001
18	0.86 ± 2.29	9.39 ± 6.90	< 0.001
Total	99.27 ± 64.84	217.64 ± 85.04	< 0.001

*SER*: spherical equivalent refractive error; *D*: diopter; *BCVA*: best corrected visual acuity; *LOCS III*: Lens Opacities Classification System III; *NC*: nuclear color; *NO*: nuclear opalescence; *C*: cortical cataract; *P*: posterior subcapsular cataract; *OSI*: objective scatter index; *cpd*: cycles per degree. *P* < 0.05 at two tails was considered to be statistically significant

**Table 2:** Characteristics, optical performance and CSF of the ERM eyes

Parameters	Stage II (*n* = 11)	Stage III (*n* = 21)	Stage IV (*n* = 12)	*P*-value
Age (Years)	61.91 ± 9.29	64.00 ± 7.47	64.83 ± 9.49	0.698
SER (D)	−0.07 ± 1.46	0.22 ± 1.18	−0.04 ± 1.22	0.772
BCVA (logMAR)	0.37 ± 0.28	0.41 ± 0.25	0.73 ± 0.19	0.001
OSI	2.04 ± 1.43	2.89 ± 1.71	4.39 ± 1.99	0.007
Strehl ratio	0.15 ± 0.03	0.11 ± 0.05	0.10 ± 0.05	0.016
Spatial frequency (cpd)				
1.5	47.36 ± 15.76	32.05 ± 19.80	16.17 ± 5.97	< 0.001
3	56.36 ± 25.46	34.48 ± 22.85	10.92 ± 6.14	< 0.001
6	45.73 ± 17.33	30.14 ± 17.62	10.67 ± 13.25	< 0.001
12	10.82 ± 8.85	3.67 ± 5.08	0.58 ± 2.02	< 0.001
18	1.82 ± 3.28	0.52 ± 1.72	0.58 ± 2.02	0.285

*SER*: spherical equivalent refractive error; *D*: diopter; *cpd*: cycles per degree. P < 0.05 at two tails was considered to be statistically significant

The ERM eyes were identified to 4 stages according to the B-scan OCT images (stage II, *n* = 11; stage III, *n* = 21; stage IV, *n* = 12). Characteristics, optical performance and CSF in the 4 subgroups of the ERM eyes were summarized in Table 2. Statistically significant differences in BCVA, optical performance and CS values were found between the 4 subgroups (all *P*<0.05). BCVA was progressively deteriorated from stage II to stage IV (P<0.001). For optical performance, values of OSI steadily increased, and values of Strehl ratio significantly decreased from stage II to stage IV (all *P*<0.05). For CSF, values at all spatial frequency, except for at the spatial frequency of 18 cpd, were also progressively decreased as the stages increased (all *P*<0.05).

The CSF as a function of the Strehl ratio was analyzed to determine any correlation between visual performance and retinal-image quality. Significant correlation between the total CS value and the Strehl ratio were found for the ERM and the fellow eyes (both *P* < 0.05). The correlation coefficient of r for the ERM and the fellow eyes were 0.60 and 0.35, respectively. In addition, BCVA significantly correlated to the total CSF (ERM eyes, *r* = − 0.53, *P* < 0.001; the fellow eyes, *r* = − 0.467, *P* = 0.002) and Strehl ratio (ERM eyes, *r* = − 0.485, *P* = 0.001; the fellow eyes, *r* = − 0.311, *P* = 0.043) in both of the ERM and the fellow eyes.

For the ERM eyes, the severity of the ERM was significantly correlated to the BCVA (*r* = 0.41, *P* = 0.010), OSI (*r* = 0.35, *P* = 0.032), Strehl ratio (*r* = − 0.35, *P* = 0.031) and the total CS value (*r* = − 0.69, *P* < 0.001) after adjusted for the grade of LOCS III NC, NO, C and P.

## Discussion

Until today, the real effect of Idiopathic ERM on the visual function is not better understood, especially on the retinal-image quality and visual performance. In the current study, ERM eyes showed reductions in BCVA, CS values at five spatial frequencies and Strehl ratio, and increases in OSI compared to the fellow eyes. In addition, the total CS value was significantly correlated to the Strehl ratio for the ERM and the fellow eyes. Furthermore, in the ERM eyes, BCVA, CS and most of the optical parameters were progressively deteriorated as the stages increased with a significant deformation of retina. To our knowledge, this is the very few study demonstrating a strong effect of ERM on the visual function, including BCVA, retinal-image quality and CSF.

The precise pathophysiology of the ERM is not completely clear, but many studies indicated that the characteristic changes of the inner and outer retinal microstructure, such as increase in the inner retinal layer thickness [17, 18], disruption of the inner segment ellipsoid zone and photoreceptor outer segments [19–21], may have association with vision loss in eyes with ERM. Govetto et al. [14] established a new OCT-based classification system identifying novel morphologic features of retina and found that the presence of continuous ectopic inner foveal layers has a significant association with negative VA prognosis. Nevertheless, the relationship between this anatomic change in inner retina with the retinal-image quality and CSF remains unknown. In our study, for the ERM eyes, the severity of the ERM was significantly correlated to the BCVA, OSI, Strehl ratio and the total CS value after adjusted for the grade of cataract, indicating that BCVA, CS and the optical parameters were progressively deteriorated as the severity of ERM increased with a significant deformation of retina.

OQAS can measure data on Strehl ratio and OSI which provides full information on optical performance, including aberrations, diffraction and scattering. The Strehl ratio, ranging from 0 to 1, is defined as the ratio between MTF area of the eye and the diffraction-limited MTF area. A lower value of Strehl ratio indicates poor optical quality with greater aberration and ocular scattering. In the current study, the results, that the Strehl ratio of the ERM eyes were lower compared to the fellow eyes, indicated a poor retinal-image quality for the ERM eyes. As demonstrated in previous studies [22, 23], interaction of light pass through the ocular media, reflected on the retina, all together influence the retinal-image quality. The OSI can quantitatively measure the intraocular scattering, which is correlated to the LOCS III system and macular thickness [15]. It indicated that OSI could be used as an index to reflect the abnormality in retina, if the crystalline lens was normal. In the current study, age-related cataract cannot be avoided because most of the patients included were elderly, but the type and severity of cataract were best matched using LOCS III slitlamp grading. Consequently, a higher OSI value found for the ERM eyes suggested that anatomic retinal change in ERM eyes would present more scattering than a relative healthy eye of a subject with the same age and refractive error. In addition, our result was consistent with previous studies [24, 25] that the Strehl ratio was lower and the OSI was higher as age increased. Also, the values of Strehl ratio and OSI for the fellow eyes in the current study were consistent with Ortiz et al.’s study [25] with similar age range (Strehl ratio, 0.17 ± 0.05 vs. 0.17 ± 0.04, respectively; OSI, 1.17 ± 1.11 vs. 1.11 ± 0.50, respectively).

Both of VA and CSF are used as psychophysical testes which will be influenced by optical [26] and neural factors [27], but the CSF can evaluate the visual function more comprehensively than does VA. VA reflects the visual function under the same and high contrast, whereas the CSF measures the threshold contrast for seeing target under different spatial frequency. Nowadays, CSF has been widely accepted and used as a sensitive measure for evaluating visual function in many researches [28–30]. In the current study, significant differences were found at all spatial frequency between the ERM and fellow eyes. In addition, significant correlation between the CSF and the Strehl ratio were found for both the ERM and the fellow eyes, indicating a correlation between visual performance and retinal-image quality. Taking into account that the results for BCVA, retinal-image quality and visual performance, such as psychophysical CSF, showed a same trend for the ERM eyes, the objective devices, such as OQAS, and F.A.C.T charts could be used to assess the visual function in patients with ERM. These assessments may also be used as operational indications for the ERM or an index assessing the prognosis of surgery for the ERM, which warrants for further clinical researches.

It should be noted that we only measured the CSF under the photopic condition in this study, because the CSF test is time consuming and requires close cooperation of the patient which is difficult task especially for old patients. Therefore, further researches of ERM considered the CSF under both the photopic and mesopic conditions are warranted.

## Conclusion

In conclusion, our result demonstrated that there is a higher ocular scattering in the ERM eyes than in the normal eyes, and this may degrade the retinal-image quality and CSF. The different level of retinal-image quality and CSF may be due to the fact that characteristic anatomic abnormality caused in the retina may change the light reflected onto the retina. Therefore, it is important to assess both of the retinal-image quality using objective device and CSF in patients with ERM. In addition, our results also indicated the feasibility of using a double-pass technique or F.A.C.T test in extensive clinical researches to assess the objective and psychophysical visual quality of ERM pathologies.

## Additional file

Additional file 1: Raw data. (XLSX 21 kb)

## Abbreviations

BCVA: Best corrected visual acuity
C: Cortical cataract
CSF: Contrast sensitivity function
ERM: Epiretinal membrane
F.A.C.T: Functional acuity contrast testing
LOCS III: Lens opacities classification system III
NC: Nuclear color
NO: Nuclear opalescence
OQAS: Optical quality analysis system
OSI: Objective scatter index
P: Posterior subcapsular cataract

## References

[1] Ng CH, Cheung N, Wang JJ (2011). Prevalence and risk factors for epiretinal membranes in a multi-ethnic United States population. Ophthalmology.

[2] Cheung N, Tan SP, Lee SY (2017). Prevalence and risk factors for epiretinal membrane: the Singapore epidemiology of eye disease study. Br J Ophthalmol.

[3] You Q, Xu L, Jonas JB (2008). Prevalence and associations of epiretinal membranes in adult Chinese: the Beijing eye study. Eye (Lond).

[4] Fraser-Bell S, Guzowski M, Rochtchina E, Wang JJ, Mitchell P (2003). Five-year cumulative incidence and progression of epiretinal membranes: the Blue Mountains eye study. Ophthalmology.

[5] Michels RG (1982). A clinical and histopathologic study of epiretinal membranes affecting the macula and removed by vitreous surgery. Trans Am Ophthalmol Soc.

[6] Schmitz-Valckenberg S, Holz FG, Bird AC, Spaide RF (2008). Fundus autofluorescence imaging: review and perspectives. Retina.

[7] Dell'omo R, Cifariello F, Dell'omo E (2013). Influence of retinal vessel printings on metamorphopsia and retinal architectural abnormalities in eyes with idiopathic macular epiretinal membrane. Invest Ophthalmol Vis Sci.

[8] Wee SW, Moon NJ (2014). Clinical evaluation of accommodation and ocular surface stability relevant to visual asthenopia with 3D displays. BMC Ophthalmol.

[9] Liao X, Lin J, Tian J, Wen B, Tan Q, Lan C (2018). Evaluation of optical quality: ocular scattering and aberrations in eyes implanted with diffractive multifocal or Monofocal intraocular lenses. Curr Eye Res.

[10] Kamiya K, Asato H, Shimizu K, Kobashi H, Igarashi A (2015). Effect of intraocular forward scattering and corneal higher-order aberrations on visual acuity after Descemet's stripping automated endothelial Keratoplasty. PLoS One.

[11] Martínez-Roda JA, Vilaseca M, Ondategui JC (2011). Optical quality and intraocular scattering in a healthy young population. Clin Exp Optom.

[12] Arden GB (1978). The importance of measuring contrast sensitivity in cases of visual disturbance. Br J Ophthalmol.

[13] Plainis S, Anastasakis AG, Tsilimbaris MK (2007). The value of contrast sensitivity in diagnosing central serous chorioretinopathy. Clin Exp Optom..

[14] Govetto A, Lalane RA, Sarraf D, Figueroa MS, Hubschman JP (2017). Insights into Epiretinal membranes: presence of ectopic inner foveal layers and a new optical coherence tomography staging scheme. Am J Ophthalmol.

[15] Artal P, Benito A, Pérez GM (2011). An objective scatter index based on double-pass retinal images of a point source to classify cataracts. PLoS One.

[16] Navarro R, Artal P, Williams DR (1993). Modulation transfer of the human eye as a function of retinal eccentricity. J Opt Soc Am A..

[17] Okamoto F, Sugiura Y, Okamoto Y, Hiraoka T, Oshika T (2012). Associations between metamorphopsia and foveal microstructure in patients with epiretinal membrane. Invest Ophthalmol Vis Sci.

[18] Okamoto F, Sugiura Y, Okamoto Y, Hiraoka T, Oshika T (2015). Inner nuclear layer thickness as a prognostic factor for METAMORPHOPSIA after EPIRETINAL membrane surgery. Retina.

[19] Kim JH, Kim YM, Chung EJ, Lee SY, Koh HJ (2012). Structural and functional predictors of visual outcome of epiretinal membrane surgery. Am J Ophthalmol.

[20] Itoh Y, Inoue M, Rii T, Hirota K, Hirakata A (2013). Correlation between foveal cone outer segment tips line and visual recovery after epiretinal membrane surgery. Invest Ophthalmol Vis Sci.

[21] Watanabe K, Tsunoda K, Mizuno Y, Akiyama K, Noda T (2013). Outer retinal morphology and visual function in patients with idiopathic epiretinal membrane. JAMA Ophthalmol.

[22] Artal P, Ferro M, Miranda I, Navarro R (1993). Effects of aging in retinal image quality. J Opt Soc Am A.

[23] Castro JJ, Jiménez JR, Hita E, Ortiz C (2009). Influence of interocular differences in the Strehl ratio on binocular summation. Ophthalmic Physiol Opt.

[24] Wang YJ, Yang YN, Huang LY, Wang B, Han YC, Yan JB (2016). Optical quality and related factors in ocular hypertension: preliminary study. J Ophthalmol.

[25] Ortiz C, Castro JJ, Alarcón A, Soler M, Anera RG (2013). Quantifying age-related differences in visual-discrimination capacity: drivers with and without visual impairment. Appl Ergon.

[26] Mutyala S, McDonald MB, Scheinblum KA, Ostrick MD, Brint SF, Thompson H (2000). Contrast sensitivity evaluation after laser in situ keratomileusis. Ophthalmology.

[27] Elliott DB, Benjamin WJ (1998). Contrast sensitivity and glare testing. Borish’s clinical refraction.

[28] Oshika T, Okamoto C, Samejima T, Tokunaga T, Miyata K (2006). Contrast sensitivity function and ocular higher-order wavefront aberrations in normal human eyes. Ophthalmology.

[29] Preti RC, Ramirez LM, Monteiro ML, Carra MK, Pelayes DE, Takahashi WY (2013). Contrast sensitivity evaluation in high risk proliferative diabetic retinopathy treated with panretinal photocoagulation associated or not with intravitreal bevacizumab injections: a randomised clinical trial. Br J Ophthalmol.

[30] Ortiz C, Jiménez JR, Pérez-Ocón F, Castro JJ, González-Anera R (2010). Retinal-image quality and contrast-sensitivity function in age-related macular degeneration. Curr Eye Res.

